# Study protocol: optimized complementary feeding study (OTIS): a randomized controlled trial of the impact of a protein-reduced complementary diet based on Nordic foods

**DOI:** 10.1186/s12889-019-6466-1

**Published:** 2019-01-31

**Authors:** Torbjörn Lind, Ulrica Johansson, Inger Öhlund, Lene Lindberg, Bo Lönnerdal, Catharina Tennefors, Olle Hernell

**Affiliations:** 1 Paediatrics, Department of Clinical Sciences, Umeå University, SE-901 85 Umeå, Sweden; 2 Department of Public Health Sciences, Karolinska Institute, Stockholm, Sweden; 3 Department of Nutrition, University of California, Davis, CA USA; 4 Semper AB, Sundbyberg, Sweden

**Keywords:** Infant food, Child nutrition physiology, Body composition, Growth, Obesity, Insulin resistance, Hypertension, Child development, Microbiota, Feeding behavior, Food preference

## Abstract

**Background:**

What we eat as infants and children carries long-term consequences. Apart from breastfeeding, the composition of the complementary diet, i.e. the foods given to the infant during the transition from breast milk/infant formula to regular family foods affects the child’s future health. A high intake of protein, a low intake of fruits, vegetables and fish and an unfavorable distribution between polyunsaturated and saturated fats are considered to be associate with health risks, e.g. obesity, type 2 diabetes and dyslipidemia later in life.

**Methods:**

In a randomized, controlled study from 6 to 18 months of age we will compare the currently recommended, Swedish complementary diet to one based on Nordic foods, i.e. an increased intake of fruits, berries, vegetables, tubers, whole-grain and game, and a lower intake of sweets, dairy, meat and poultry, with lower protein content (30% decrease), a higher intake of vegetable fats and fish and a systematic introduction of fruits and greens. The main outcomes are *body composition* (fat and fat-free mass measured with deuterium), *metabolic and inflammatory biomarkers* (associated with the amount of body fat) in blood and urine, *gut microbiota* (thought to be the link between early diet, metabolism and diseases such as obesity and insulin resistance) and *blood pressure*.

We will also measure the participants’ energy and nutrient intake, eating behavior and temperament through validated questionnaires, acceptance of new and unfamiliar foods through video-taped test meals and assessment of cognitive development, which we believe can be influenced through an increased intake of fish and milk fats, notably milk fat globule membranes (MFGM).

**Discussion:**

If the results are what we expect, i.e. improved body composition and a less obesogenic, diabetogenic and inflammatory metabolism and gut microbiota composition, a more sustainable nutrient intake for future health and an increased acceptance of healthy foods, they will have a profound impact on the dietary recommendations to infants in Sweden and elsewhere, their eating habits later in life and subsequently their long-term health.

**Trial registration:**

NCT02634749. Registration date 18 December 2015.

## Background

Early feeding affects health and development later in life [1]. The positive effects of breastfeeding are well known [2], but less is known about how different types and timing of introduction of complementary foods, i.e. the foods used during the transition from breastfeeding or formula-feeding to family foods affect short- and long-term health [3]. From around 4–6 mo. of age eating habits of infants start to change from a liquid diet of breast milk or infant formula to a more diversified diet, mixing breast milk or formula with increasing amounts of solid foods, ending with the child eating the same food as the rest of the family. During this time of complementary feeding, demands for energy and nutrients remain high, especially during the second half of the first year of life. We and others have shown a link between protein intake during this time period and later risk for overweight [4, 5]. Current dietary recommendations for infants and children suggest lower protein intake compared to previous guidelines [6]. Further, we and others have shown that higher intakes of polyunsaturated fatty acids and lower intakes of saturated fatty acids in the diet of infants improve the blood lipid profile and lower blood pressure, and that these influences track into childhood [7–10]. These polyunsaturated fatty acids, especially in the long-chain form (LCPUFA) found in fish and fish oils have in some studies also been associated with lower risk of asthma, atopic disease and infections during infancy and childhood [11–13].

The composition of the complementary food is important also from the perspective of flavour, texture and presentation [14]. Already the foetus experiences different sensations of flavour through the amniotic fluid, reflecting what the mother eats [15]. Through breast milk, this exposure continues as infants encounter different flavours in the milk, depending on the types of food the mother consumes [15]. These processes prime the newborn infant to accept the same types of foods as the mother usually takes, including flavours outside the normal preferences of the infant, i.e. sweet, salty and fatty [16]. Further, the acceptance of different textures of foods changes during infancy. As the oral motor skills of the infant develop, the child is able to handle increasingly lumpy and solid foods. A normally developing infant will have periods of greater acceptance of novel foods both in terms of taste and texture, usually coinciding with the time when complementary foods are introduced [17]. However, this window of opportunity to introduce new foods begins to close, usually around 10–12 mo. of age, when the child grows increasingly suspicious of unfamiliar tastes, i.e. develops food neophobia, but also, if solids have not been introduced at this time, to the texture of common food items. This reluctance to try new food items particularly applies to fruits and vegetables, which have a bitter and sour tastes and need repeated exposures to be learned to be appreciated [18]. Numerous studies have shown that fruits and vegetables are an important part of healthy eating habits during all ages [19], but that the actual intake of these food items among young children is low and the best ways to increase the intake largely unknown [20]. Population-based studies from Sweden and elsewhere have shown that the dietary intake of children is not satisfactory from a public health perspective, with intakes of refined sugars, fatty foods and salt being too high and intakes of fruits and vegetables being too low [21]. A recent report on food habits of Swedish adults from the National Food Agency found the worst food habits among young adults age 18–30 years, especially among young women [22]. This is of concern as this is the age when people start families and possibly transmit their poor eating habits to their children.

The New Nordic Food programme [23] is an initiative that highlights the health, gastronomic and sustainability aspects of foods produced within the Nordic region [24]. The overall aim of the diet is to: a) allow a higher energy intake from plant foods and a lower energy intake from meat, b) consume more sea and lake foods and c) consume more foods from the rural countryside [25]. Compared to the regular Swedish diet, the Nordic diet (ND) stresses higher intakes of regionally produced fruits, berries, vegetables, tubers, and legumes as well as higher intakes of whole-wheat, vegetable fats and oils, fish and egg, and lower intake of sweets, desserts and dairy, meat and poultry products, but at the same time being in line with current dietary recommendations [26]. In adults, studies on ND have shown beneficial effects on weight and metabolic and cardiovascular disease biomarkers of the same magnitude as the Mediterranean diet [27, 28] as well as on total mortality [29]. The diet has also been evaluated in a large intervention study among Danish school children aiming to improve dietary intake, nutrient status, growth, early risk biomarkers for adult chronic disease, well-being and cognitive function [30, 31]. The results show an improved dietary intake including higher intakes of fish and vegetables [31] and metabolic effects such as a decrease in mean arterial blood pressure, decreased total cholesterol and improved insulin resistance [32, 33].

Differences in eating behaviour among infants and children is an issue that influence the risk of future obesity [34]. These patterns evolve already in infancy, where genetic predisposition, e.g. sensitivity to bitter taste [35] combine with natural responses to foods and flavours, actual food exposure and parental feeding practices to form the eating style of the individual [36]. Behavioural patterns in childhood that have specifically linked to later obesity include internal factors such as high enjoyment of food, high food responsiveness, emotional overeating and low responsiveness to internal satiety cues, e.g. high eating speed and low satiety sensitivity and external factors such as parental restriction. These behaviours are important to include when examining effects of different diets on later health outcomes and can be measured by validated questionnaires [37–39].

The nutrient intake of the infant affects its development, for example an adequate iron intake during the second half year of life increases psychomotor development compared to infants not receiving iron supplementation [40]. Systematic reviews of studies on supplementation with LCPUFAs to healthy term infants have failed to show effects on cognitive development measured with the standardized tests such as Bayley Scales of Infant Development (BSID) [41, 42]. However, in even quite small interventions of LCPUFAs during infancy, using more specific tests of brain development has shown positive both short- and long-term effects [43, 44]. Also, a recent randomized, controlled intervention study supplementing infant formula with bovine milk fat globule membranes (MFGM) found a significant effect on cognitive development [45]. It is of note, that the methodology to measure cognitive and psychomotor development following nutritional interventions in infancy using the BSID has recently been challenged, calling for more sensitive methods, including for example attention free play [46].

The gut microbiota has attracted increasing attention during the last several years as a key player in the pathophysiology of chronic disease [47, 48]. Effects on faecal microbiota composition has been a proposed pathway linking early dietary intake with later metabolic outcomes [49] and the development of overweight and also allergic diseases [50]. With the transition from breast-feeding to complementary feeding the gut microbiota undergoes significant changes where correlations have been found between infancy body mass index (BMI) and the faecal flora [51].

In the present study, we draw from the research outlined above, aiming to improve body composition, metabolic markers, cognitive development, blood pressure, food acceptance and the faecal microbiota composition of young children by using a protein-reduced, ND as a basis for complementary feeding.

## Methods/Design

The overall aim of the study is to compare the effects of a protein-reduced, Nordic diet (ND) portfolio intervention on body composition, metabolic and inflammatory markers, blood pressure, cognitive development, food acceptance and faecal microbiota composition to conventional complementary feeding from 6 to 18 months of age.

More specifically, we will investigate if a complementary diet:

- Lower in protein (total reduction 30%) from 6 to 18 mo. of age will affect growth, body composition, i.e. reduce total body fat mass and improve metabolic markers, i.e. decrease plasma (P-) levels of insulin, glucose and insulin-like growth factor 1 (IGF-1)
- Based on Nordic foods from 6 to 18 mo. of age will affect growth, body composition, i.e. reduce body fat mass and improve metabolic markers and the faecal microbiota composition, i.e. a microbiota composition associated with less obesity and inflammation
- With a modified fat intake, i.e. a higher intake of long-chain polyunsaturated fatty acids and milk fats, including MFGM will improve body composition, blood pressure, metabolic markers including erythrocyte fatty acid composition, cognitive development and the faecal microbiota composition
- With a systematic introduction of plant foods from the Nordic diet during weaning will increase the acceptance of a larger variety of fruits, berries and vegetables into the diet of the young child at 12 and 18 mo. of age

Primary and secondary outcomes of the study and variables collected on adherence and background factors are shown in Table 1.

**Table 1:** Primary and secondary outcomes and adherence and background factors collected the Optimized complementary feeding study

Primary outcomes	
Body composition	Anthropometry (body weight, body length, head circumference) Fat and fat-free mass (deuterium-labelled water)
Biomarkers of metabolic function	P-insulin, P-glucose, P-IGF-1, P-lipids and lipoproteins, P-fatty acids and erythrocyte membrane fatty acids including LCPUFAs (DHA, EPA, ARA), metabolomics (plasma and urine) and lipidomics (plasma), markers of oxidatative capacity, hsCRP
Faecal microbiota composition	Obesity-promoting microflora
Blood pressure	
Secondary outcomes, adherence and background factors	
Dietary intake	
Biomarkers of adherence	Haemoglobin, iron status, P-urea, vitamins C, A, E, carotenoids, folic acid, C15 and C17 fatty acids
Food acceptance	Videotaped test meal
Eating behaviour	CEBQ, CFQ, COC, bitter taste gene *TAS2R38*
Temperament	BBQ and TBQ
Cognitive development	Attention free play and ASQ
Allergy and atopic manifestations	
Symptoms registration	
Background factors	Child’s neonatal history, early nutrition, past and present illnesses, family composition, mother’s pregnancy history, education, employment, smoking habits, history of chronic illnesses, father’s education, employment, smoking habits, history of chronic illnesses

### Participants and recruitment

Through invitation-letters sent to all infants in Umeå municipality at 3 mo. of age, healthy, full-term singletons will be recruited. When parents consider it appropriate to introduce complementary foods into the diet of their child, but no later than at 6 mo. of age, the child will be randomly allocated to one of two study groups, i.e. the Nordic diet group (intervention, ND) or the Regular diet group (control).

#### Inclusion criteria

- Healthy, singleton infants 4–6 mo. of age
- > 37 weeks of gestation at birth
- Birth weight > 2500 g
- Available throughout the study period, i.e. the participant will remain in the study area (Umeå municipality) and will not commence childcare outside the home during the extent of the study, i.e. until 18 mo. of age.
- Parents or legal guardians are able to give written informed consent to participation in the study.

#### Exclusion criteria

- Children with chronic illnesses that will affect feeding or growth, including food allergies or intolerance to study products
- Intake of any complementary food at recruitment
- Use of supplements or medications that will affect the study outcomes
- Iron deficiency (Hb < 105 g/L, serum (S-) ferritin < 12 μg/L) or any other biochemical abnormality discovered at the baseline examination that needs medical attention after decision by the study physician.
- Repeated non-adherence to key study procedures including anthropometric measurements, allergy, eczema and symptoms registrations.

### Informed consent

A written Informed Consent from the subject’s parents/caregiver is an inclusion criterion without which the subject cannot be included in the study.

### Randomisation, blinding, coding and data management

**Fig. 1:**
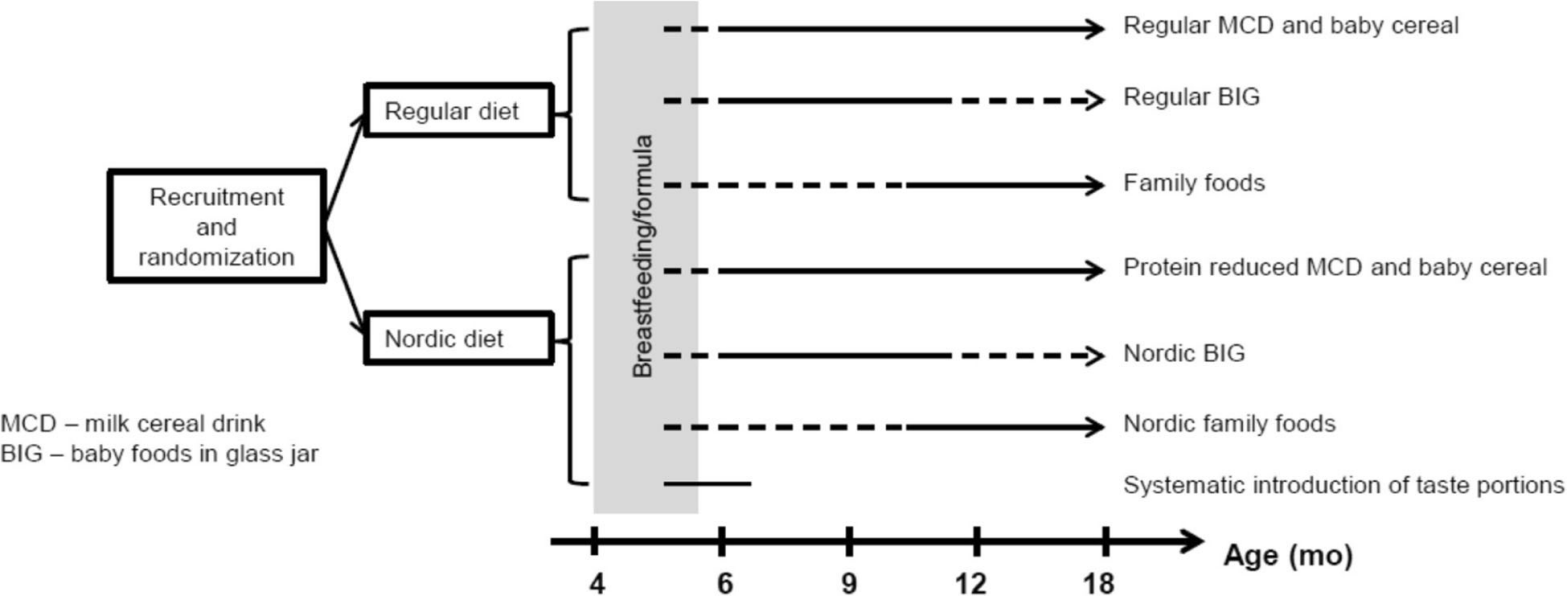
Intervention and data collection in the OTIS study

The participants will be randomized at recruitment to either the Nordic diet (ND, intervention) or the Regular (control) group (Fig. 1). Group affiliation will be blinded for participating families and the researchers. Boys and girls will be randomised separately in blocks of 10 from a pre-printed, computer-generated list available to the research nurses, who will enter in participants consecutively. Each participant will be given a unique study identification number. The identification number together with the date of birth will serve as identification on all forms, questionnaires, measurements and as participant identifier in the study database. The key identifying each individual with the unique identification number will be kept in a locked safe to which only researchers and administrative staff at Paediatrics, Department of Clinical Sciences has access. All data collected will be regarded as confidential and stored electronically in coded format according to the Swedish Personal Data Act (1998:204) and the European Union General Data Protection Regulation (EU 2016/679), but paper copies of forms will be kept as well.

### Study products

All participants will be supplied with study products free of charge. In the ND group, these will be specially prepared, protein-reduced, age-adjusted milk cereal drinks (MCDs), baby cereals and baby milk and commercially available baby foods in glass jars (BIG). The control group will be offered commercially available, age-adjusted MCDs, baby cereals, baby milk and BIG. Semper AB, Sundbyberg, Sweden, will provide all products.

The participants will be provided a month’s supply of study product at baseline and subsequently the families will come to the Paediatric Research Unit to collect more products as needed but not more than for one month at a time.

### Introduction of complementary feeding

The switch from the liquid diet of the young infant to the mixed diet of the older infant or toddler is a gradual process. First, when the infant starts showing interest in other foods the parent or caretaker offers small taste portions of various simple foods to take benefit of the infant’s curiosity. These small titbits, in Sweden usually served as purees or mashes of various vegetables, are provided as a way to familiarise the infant to new flavours and consistencies of foods which the infant will eat in the future, but they contribute little to the total energy and nutrient intake of the infant, which at this time is covered by breast milk or infant formula. Later, as the infant grows and develops many parents start substituting one or more breast- or formula-feeds with more and more solid food.

The ND group will be given recipes for fruit, berries and vegetable purees and instructions how to prepare these recipes at home. The main ingredients will be fruits, berries and vegetables which grow and are available in the Nordic region [26]. The dishes will be given as small taste meals to the participating infant when the parents feel it prudent to start offering the infant other foods besides breast milk or formula and the infant is deemed ready by the parent. This time-point is to be chosen by the parents themselves. When the time has come for taste meals, the instructions to the parents will be that they should systematically introduce eight different fruits, berries and vegetables, each one given at least 3 times daily for three consecutive days whether or not the child immediately appreciates the flavour/consistency the first time. After the first 3 days, the parent will switch to another fruit, berry or vegetable until the different ingredients have been sampled. In total, there will be a maximum of 72 exposures during 24 days.

The control group will be given the standard, oral and written advice that is given from well-baby clinics to parents when beginning with small taste meals based on national guidelines [52].

### Study design and interventions

After recruitment the participating child will be randomized to either a regular (control) diet or the experimental Nordic diet (ND).

#### The Nordic diet group

The Nordic diet group will experience three interventions (Fig. 1): i) a systematic introduction of taste portions, ii) milk cereal drinks (MCD), porridge and baby milk with reduced protein content, and iii) homemade and industry manufactured (BIG), protein-reduced main meals with a predominance of Nordic ingredients.

1. When the parents have made the decision that their child should start with complementary foods, the ND group will be given instructions to systematically introduce four different fruits and berries (apple, raspberry, buckthorn/lingonberry, cranberry) and four vegetables (green peas, cauliflower, turnip, daikon), each one given at least 3 times daily for 3 consecutive days whether or not the child likes the flavour/consistency the first time or not. In total, there will be a maximum of 72 exposures during 24 days.
2. When the parents start to complement the baby’s diet with MCD and/or porridge, the participants will be given protein-reduced products. The families will be instructed to use these products at their own discretion and they will have an offer to continue with these study products for as long as the child remains in the study.
3. When the child is ready for a larger variation in meals, the ND group will be given recipes for main meals (lunch, dinner) and BIG with predominantly Nordic ingredients. The families can use these recipes and BIG as they see fit, but we will recommend that the families use the recipes for home-made food at least once daily.

#### The regular diet group

The regular diet group will function as control for to the ND group (Fig. 1). The participants will be given the current advice on infant feeding issued by the Swedish National Food Agency [52]. They will also be given regular, commercially available MCD, porridge, baby milk and BIG. No advice or recipes for meals will be given apart from the current recommendations.

### Parental support

Parents with children in the ND group will be given support to continue following the dietary intervention. They will be invited to participate in a closed Facebook™ group. In this forum parents can discuss with a study dietician and among themselves, sharing experiences. Parents will also have instructional videos on the preparation of certain study foods available within the group setting. Participants in the group will share experiences and other information at their own discretion. No data will be collected through this group, but the group may function as a way to inform the families that participate in the group about upcoming study events and remind participants to send in questionnaires, etc.

Both the intervention and the control groups will get monthly telephone calls from a research nurse or a study dietician as part of the regular data collection (see below).

### Data collection

Main data collection points will be at baseline, i.e. when the child is between 4 and 6 mo. of age, and at 9, 12 and 18 mo. of age. At these time-points, the participants will come to the Paediatric Research Unit for measurements and sampling (Fig. 1). Questionnaires and forms will be available electronically or on paper. Electronic questionnaires will be administered through Texttalk Websurvey, a tool provided by Umeå University for electronic surveys. The questionnaires and forms will be filled out prior to the main data collection points. Questionnaires on paper will be collected at these times. All forms, either electronic or paper will be checked for completeness at the time when they are returned to the researchers. Incomplete or possibly erroneous data will be double-checked with the participating families through telephone follow-up. All data will be entered and stored electronically in a secure database provided by Umeå University. Paper copies of questionnaires and measurement protocols will be stored in accordance with University rules in a safe archive to which only researchers and administrative staff at Paediatrics, Department of Clinical Sciences has access.

#### Sociodemographic data

At baseline, information will be collected on family size (number of sibling, household structure), living conditions, country of birth, parental education, occupation and smoking habits, chronic diseases in the family history (allergies, atopic diseases, diabetes type 1 and 2, obesity, hypertension, stroke, cardiovascular disease, cancer), details on the birth of the participating child (including a request to collect the birth document from the mother’s health record), and the health and feeding pattern since birth of the participating child.

#### Adherence

The research nurse or dietician will contact the participating families monthly by telephone to check adherence to the protocol, study related problems, consumption and supply of study products and the symptoms registration form. Data from the telephone interview will be registered electronically.

#### Anthropometry and blood pressure

Anthropometric measurements will be taken at baseline (4–6 mo. of age), 9, 12 and 18 mo. of age at the Paediatric Unit (Department of Clinical Sciences, Umeå University, Umeå, Sweden) by experienced paediatric research nurses in accordance with standardized procedures [53]. Nude weight will be measured to the nearest 0.1 kg with electronic baby scales (Seca 727, Bromma, Sweden). Recumbent length will be measured to the nearest 0.1 cm with a paediatric length board (CMS Weighing Equipment Ltd., London, UK). Head circumference will be measured to the nearest 0.1 cm with non-stretchable tape for infants (Seca 212, Germany). All measurements will be taken in triplicate and the mean value will be recorded.

Blood pressure will be measured with the child sitting in the parent’s lap at baseline (4–6 mo. of age), 12 and 18 mo. of age using a Carescape Dinamap V100 monitor (GE Healthcare AB, Sweden) and age-appropriate blood pressure cuffs.

At baseline, the weight, height and blood pressure of the parents will be measured.

#### Body composition

Total body water (TBW) as a measure of body composition (3 compartments model including total body water, fat and fat-free mass) will be determined at 12 and 18 months of age. TBW will be assessed using deuterium (^2^H_2_O) according to the procedure advised by the International Atomic Energy Agency [54] in collaboration with the MRC Human Nutrition Research Laboratory, Cambridge, UK. A pre-dose urine sample will be collected by placing an absorbent pad (Bastos Viegas, Penafiel, Portugal) in the diaper of the infant. Each infant will then be given an oral weighed dose of deuterium of 1 g/kg. Post-dose urine samples will be collected at home once daily for five consecutive days with dates and times recorded for all samples using absorbent pads as described above, omitting the first urine portion of the day. Each collected pad will be stored at − 18 °C. The pads will then be taken to the Paediatric research facility at Umeå University Hospital, thawed and the urine content extracted using a press, collecting the urine in glass bottles. The glass bottles will be stored at − 20 °C until transported to MRC Elsie Widdowson Laboratory, Cambridge, UK for analysis.

#### Dietary intake, eating behaviour and food acceptance

Dietary intake will be assessed using a 5-day food record. This methodology has been extensively used in this age group by the researchers [7, 55]. On four occasions, at baseline (4–6 mo. of age), 9, 12 and 18 mo. of age, the parents will be asked to register the participating child’s intake of foods and drinks during consecutive 5 days. Parents will be instructed how to use record sheets and household measures to quantify and describe their infant’s dietary intake. Previous research has found that parents tend to underestimate energy intake in this age group using only food records [56]. In order to improve estimates of energy and nutrient intake, the parents will also be asked to photograph the child’s meal portions during the 5-day food record, just before the meal is served and right after, taking 2 photos each time from perpendicular angels. The parents will send the photos electronically to a study-specific cloud service provided by Umeå University. In a separate sub study, this methodology will be validated using doubly labelled water.

Eating behaviour will be assessed by three recently validated, Swedish questionnaires, the Children’s Eating Behaviour Questionnaire (CEBQ) [57], the Child Feeding Questionnaire (CFQ) [58] and the Covert and Overt Control Questionnaire (COC) [59]. The questionnaires will be administered in electronic form (Texttalk Websurvey), the CEBQ together with the 5-day food record and the CFQ and COC at baseline.

A videotaped test meal to assess food and taste acceptance will be administered at 12 and 18 mo. of age. The video recording will take place in the home of the participant. During the meal, the parent will serve a prepared study meal (fruit-flavoured yoghurt, fruit-flavoured milk and a cracker with a vegetable spread) to the child. The meal will be videotaped and later assessed for acceptance in collaboration with associate professor Lene Lindberg, Karolinska Institute, Stockholm, Sweden.

#### Biochemical data

Venepuncture blood samples will be collected at baseline (4–6 mo. of age), 9, 12 and 18 mo. of age. Samples will be collected by experienced paediatric research nurses at the Paediatric Research Unit (Department of Clinical Sciences, Paediatrics, Umeå University, Umeå, Sweden). A local anaesthetic cream will be applied prior to blood sampling. The total collected blood volume will be less than 3 ml/kg per 24 h [60]. Blood for immediate analysis (complete blood count, iron status, P-urea, hsCRP) will be taken off and sent to the Department of Clinical Chemistry, Umeå University Hospital. The remaining blood will be centrifuged, the plasma removed and frozen at − 80 °C until further analyses. At baseline, blood will be collected from the parents for analyses of iron status (complete blood count, serum (S-) ferritin, S-Fe and S-transferrin) and metabolomics. Urine will be collected by placing an absorbent pad (Bastos Viegas, Penafiel, Portugal) in the diaper of the infant at baseline (4–6 mo. of age), 12 and 18 mo. of age for analyses including metabolomics, lipidomics and oxidative status. The urine will be stored at − 20 °C until further analyses. Metabolomics and lipidomics will be done in collaboration with the Swedish Metabolomics Centre, Umeå and prof. Carolyn Slupsky, Department of Nutrition, University of California, Davis USA (our group collaborates with both platforms). Any remaining blood or urine will be stored in the Biobank of the Department of Clinical Sciences, Paediatrics, Umeå University, Sweden.

#### Faecal and saliva samples

Faecal samples will be collected at baseline (4–6 mo. of age), 12 and 18 mo. of age for analysis of microbiota composition. Analyses of the faecal microbiota composition will be done at the Paediatric Research Laboratory, Department of Clinical Sciences, Paediatrics, Umeå University, Umeå, Sweden. Any remaining faecal material will be stored in the Biobank of the Department of Clinical Sciences, Paediatrics, Umeå University, Sweden.

Saliva samples will be collected for genetic analyses. These will be done in collaboration with Professor Stefan Johansson, The Center for Medical Genetics and Molecular Medicine, Haukeland University Hospital, Bergen, Norway. The analyses which include genome wide association studies (GWAS) as well as specific analysis of the bitter taste gene *TAS2R38* [61]. Homozygotic presence of the bitter-taste sensitive haplotype has been associated with lower intake of vegetables and higher intake of sweets in Finnish adults [62], but the effect on dietary intake during childhood is less clear [63]. In a separate sub-study we will explore genetic factors associated with weight development and the effects of complementary foods.

#### Developmental assessment

Child temperament, which may influence food acceptance will be assessed with the Swedish versions of the Baby Behaviour Questionnaire (BBQ) at baseline (4–6 mo. of age) and the Toddler Behaviour Questionnaire (TBQ) at 18 mo. of age [64]. To assess general cognitive and psychomotor milestones, the Ages and Stages Questionnaire (ASQ) [65] will be administered at baseline (4–6 mo. of age) and again at 12 and 18 mo. of age as a measure of general development.

A videotaped single object free play task [66, 67], as a measurement of cognitive development in relation to the nutritional intervention will be administered at 12 and 18 mo. of age at the time of the blood sample and anthropometric measurements. The test will be administered at the Paediatric Research Unit (Department of Clinical Sciences, Paediatrics, Umeå University, Umeå, Sweden). The videotapes will be assessed for cognitive development in association with associate professor Lene Lindberg, Karolinska Institute, Stockholm, Sweden. This methodology has been used when assessing cognitive effects of LCPUFAs in Danish infants [68].

#### Data on atopic disease and allergic manifestations

Family history of allergies, atopic diseases and asthma among parents and siblings will be collected at baseline. At 9, 12 and 18 mo. of age the participants will be asked for symptoms of allergy, asthma and atopic disease, and the research nurse will use a score sheet to assess eczema [69].

#### Symptoms registration

The participants will register days with symptoms of gastrointestinal disturbances (diarrhoea, constipation, vomiting), fever (≥38.0 °C), respiratory signs (cough, difficult breathing, coryza), sleep disturbances, skin symptoms, ear pain, teething, vaccinations, an estimate of the current appetite (decreased, normal, increased), if the child is being given medications and if the child has been seen by a doctor (because of illness) or if the child has been admitted to hospital. The forms will be filled out on paper and returned monthly to the researchers. At the monthly follow-up by a research nurse or dietician, these forms will be checked for completeness.

### Procedures

The baseline and 9, 12 and 18 mo. visits will take place at the Paediatric Research Unit at the Department of Clinical Sciences, Paediatrics, Umeå University Hospital.

At baseline, the researchers will establish that the participant fulfils all the inclusion criteria and none of the exclusion criteria, written informed consent will be collected if this has not been collected earlier and the parents will be given spoken and written information about the study and time to ask questions on the study procedures. The parents will also be given information on how to complete questionnaires and forms, whether on-line or on paper, how to upload photos and, if the participants are randomized to the ND group, how to access the on-line support forum, i.e. the closed Facebook group of the study. The participants will then be assessed according to the Figure including drawing blood samples and provided with a month’s supply of study products and recipes.

At ages 7 and 8 mo., the research nurse or dietician will have a telephone follow-up with the participants checking adherence, possible study related problems, supply and consumption of study products and symptoms registrations.

At age 9 mo. the participants will again come to the Paediatric Research Unit for measurements according to the Figure.

At ages 10 and 11 mo. the research nurse or dietician will have a telephone follow-up with the participants checking adherence, possible study related problems, supply and consumption of study products and symptoms registrations.

At 12 mo. of age, the participants will again come to the Paediatric Research Unit for providing samples according to the Figure. Further, the research nurse will do a home visit for anthropometric measurements, administration of the deuterium labelled water and the two videotaped tests, i.e. food acceptance test and the attention free play test.

At ages 13–17 mo., the research nurse or dietician will have a telephone follow-up with the participants checking adherence, possible study related problems, supply and consumption of study products and the symptoms registrations.

Finally, at 18 mo. of age, the participants will come for the final measurements of the study according to the Figure. The research nurse will again do a home visit for anthropometric measurements, administration of the deuterium labelled water and the two videotaped tests, i.e. food acceptance test and the attention free play test.

### Adverse events

An adverse event (AE) is any undesirable medical occurrence in a subject administered a study product, regardless of its cause. An adverse event can therefore be any unfavourable and unintended sign (including an abnormal laboratory finding), symptom, or disease temporally associated with the use of a study product, whether or not considered related to the study product. All adverse events, including observed or volunteered problems, complaints, or symptoms will be recorded on the Symptoms registration form. Each adverse event is to be evaluated for duration, intensity, and causal relationship with the study product or other factors. The participants will be instructed to report any AE that they experience to the researchers through the Symptoms registration form and by contacting the researchers. The research nurse will assess AE at each visit, i.e. 9, 12 and 18 mo. of age and at all monthly telephone follow-ups and report to the principal investigator (TL).

### Withdrawal

The participants may withdraw from the study at any time by i) withdrawing consent, ii) through non-compliance (repeated failure to comply with study procedures) or if iii) the participant experiences adverse events requiring the participant to withdraw on advice of the research physician or by his/her own accord. If possible the researchers will explore the specific reasons why consent was withdrawn, type of non-compliance and which specific AEs that caused withdrawal. All reasons for withdrawal will be recorded. Participants who withdraw will not be replaced.

### Power calculation and statistical analyses

The primary outcome of the study is body composition, i.e. fat mass measured by TBW. The body fat mass is on average 2.9 ± 0.6 kg at 12 mo. of age. In order to detect a difference in body fat of 0.24 kg between the intervention (ND) and control groups at 12 mo. of age (α = 0.05, power = 80%) with an attrition of 20%, we will need 125 participants per group.

Comparisons of differences in main outcomes between the ND and control groups will be the focus of the statistical analyses. The primary outcomes will be analysed according to the intention-to-treat principle when comparing the two study groups, but per protocol analyses will also be done. Both parametric and non-parametric tests will be used depending on the distribution of data. Possible effect modifiers and confounders will be included in the analyses as needed to explain group differences. Data on dietary intake will be converted to energy and nutrients through nutrient calculation software (Dietist NET, Kost och Näringsdata AB, Bromma, Sweden).

## Discussion

The early diet has long-lasting effects on human health. Apart from breastfeeding, little is known about which infant diets would confer long-term health benefits. ND has several potential advantages in this respect, and studies in adults have shown favourable effects on metabolic- and cardiovascular disease markers and mortality similar to those gained through the Mediterranean diet. The present study, using ND as the basis for complementary foods but also reducing the total protein intake, improving fatty acid composition and expanding the introduction of fruits and vegetables into the diet of young children will add vital information in the quest to prevent obesity, dyslipidaemia, insulin resistance, type 2 diabetes, hypertension and cardiovascular disease. If the objectives of the study are reached it will thus serve to improve dietary recommendations to infants in Sweden and worldwide. Strengths of the study include individual randomization of the participants, use of well-validated methods of data collection and analysis and close follow-up and support to the participants through dedicated research staff. Possible limitations include difficulties of the families accepting and adhering to the ND, which may lead to higher drop-out rate in the intervention group. The focus on ND may also skew recruitment away from non-Nordic participants. However, this will most likely not affect the outcomes.

Through early intervention, i.e. already when complementary foods are introduced into the diet of the infant, we believe that we can initiate healthy food preferences from the earliest date, thus optimizing the long-term public health benefits. By adopting the concept of ND we will also add gastronomical aspects to the development of optimal complementary foods as well as support regional food production and contribute to a sustainable environment.

### Trial status

Recruitment started April 2015 and ended February 2018 with 250 participants included. Data collection will continue until February 2019.

## Abbreviations

AE: Adverse event
ASQ: Ages and Stages Questionnaire
BBQ: Baby Behaviour Questionnaire
BIG: Baby foods in glass jars
BMI: Body mass index
BSID: Bayley Scales of Infant Development
CEBQ: Children’s Eating Behaviour Questionnaire
CFQ: Child Feeding Questionnaire
COC: Covert and Overt Control Questionnaire
GWAS: Genome wide association studies
LCPUFA: Long-chain polyunsaturated fatty acids
MCD: Milk cereal drink
MFGM: Milk fat globule membrane
ND: Nordic diet
P: Plasma
S: Serum
TBQ: Toddler Behaviour Questionnaire
TBW: Total body water
